# Professional Dishonor

**Published:** 1888-10

**Authors:** E. Van Goidtsnoven

**Affiliations:** Atlanta, Ga.


PROFESSIONAL DISHONOR.—We give space below to a letter from one of the ward physicians of Atlanta. The discourtesy therein criticised is mortifying to all members of the profession who regard its honor and its integrity before the world. The desertion of a dying patient with a view of throwing the case into the hand of a brother practioner is certainly most unfair and dishonorable. Such conduct deserves the scorn and contempt of all good men in the profession. Of all classes in the world medical men should be true to each other. To call a consultation in extreme, and even hopeless cases, is legitimate and honorable, and when thus called we should go and sustain as far as possible the brother in charge, but to desert the case on a false pretext, or get drunk, as we have often seen done, in order to throw it into the hands of another physician, is dishonorable in the extreme. Let us in all things be kind, liberal and just to each other. It is honorable and right; as a matter of policy it is wise and best, and in the end its results, both moral and financial, will prove far better for all parties.

ATLANTA, GA., September 22, 1888.

*Editors Southern Medical Record:*

Diplomas do not make doctors; neither do they make gentlemen. I beg you will pardon me if I solicit the use of your valuable columns to enter a most solemn protest against the acts of some medical men. The old maxim of Confucius: "Do unto others as you would be done by," is to them what honor is to the brute creation, and the Code of Ethics is no more binding on their sense of proper relation with their colleagues than is the Derby on their shallow brains. The number of mean acts that these professionals are culpable as well as capable of is simply incalculable. I will here cite one of those, yea, *a pluribus unum*, which has repeatedly fallen under my observation. No doubt the guilty parties on reading this will call it a joke on the city physician in order to pacify their conscience. For my part, I have no hesitancy in pronouncing it the proceeding of a man born without the instinct of a gentleman. I am, as you well know, one of the city physicians of Atlanta, and as such, required to wait on all the sick paupers or indigents of the Second Ward. There hardly passes a week that I am not called upon to wait on one of those unfortunates, white or black, whose pocket and even health has been drained by these medical sharks; and when the money is no longer forthcoming, they are GENTLY and COMPASSIONATELY turned over to the city physician for subsequent and gratuitous attention. It not rarely occurs that having undertaken to treat a case for a considerable time in the expectancy of a fee, they find an excuse for abandoning it, when their patient is about to die, especially if the latter be poor, and the city physician after having waited on a moribund, is unavoidably required to issue the death certificate! Now, these are facts which can be corroborated by my colleagues of the other wards! How kind and charitable to the patient! What an act of PROFESSIONAL COURTESY!

I do not ask for a remedy, for there is nothing more impervious than a callous heart and a calculating mind! But I simply wish to expose an evil to society and a stain on the profession. Very respectfully,

E. VAN GOIDTSNOVEN, M.D.

